# The Importance of Inter-Species Variation in Traumatic Brain Injury-Induced Alterations of Microglial-Axonal Interactions

**DOI:** 10.3389/fneur.2018.00778

**Published:** 2018-09-20

**Authors:** Karen M. Gorse, Audrey D. Lafrenaye

**Affiliations:** Department of Anatomy and Neurobiology, Virginia Commonwealth University, Richmond, VA, United States

**Keywords:** traumatic brain injury, microglia, axonal injury, microglial-neuronal interaction, rat, micro pig, species differences

## Abstract

Interactions between microglia and neuronal components are important for normal CNS function. They are also associated with neuroinflammation and many pathological processes and several studies have explored these interactions in terms of phagocytic engulfment. Much progress has also been made in understanding the consequences of chronic neuroinflammatory changes following trauma. However, little is known about acute alterations to these physical non-phagocytic microglial-neuronal interactions following traumatic brain injury (TBI), and particularly to what degree these post-injury interactions may be influenced by the animal species utilized in pre-clinical models of TBI. To investigate these problems, we evaluated the physical interactions between microglia and injured axons acutely (6 h and 1 day) following central fluid percussion injury (cFPI) in both rats and micro pigs. The physical interactions between Iba-1+ microglia and either normal MBP+ myelinated fibers or APP+ injured axonal swellings in the thalamus were assessed following injury or sham via quantitative image analysis of 3D confocal micrographs. The results indicated that the physical interactions between microglia and injured axonal swellings decreased by nearly half in rats 6 h following cFPI but was consistent with sham control at 1 day post-cFPI. This reduction was also observed in non-injured intact fibers at both timepoints following TBI in the rat. Microglial process interactions with injured axons in the micro pig, however, increased nearly 2-fold compared to interactions with intact axonal segments 1 day post-cFPI. This study shows that the species utilized for *in vivo* pre-clinical studies influences the manner in which microglial-axonal interactions change following TBI. These species differences can be leveraged to further our understanding of the mechanisms involved in microglial process convergence and how these neuro-immune interactions alter the progression of axonal injury following TBI.

## Introduction

Neuroinflammation and the interactions between microglia and neuronal components are important for normal CNS function. Within the healthy brain, processes of ramified surveying microglia make frequent contacts onto neuronal segments, including soma, axons, and synapses (1–4). Prolonged physical interactions between microglial processes and synaptic boutons result in synaptic pruning during normal development (1). Active microglia are integral for proper synapse formation, appropriate learning/memory, and are a potential mediator of regeneration through the secretion of neurotrophins (5–9). Active microglia also play the vital role of clearing debris from degenerating neurons via phagocytosis (9, 10). These phagocytic activities have been the primary focus of physical interactions between microglia and axons following injury.

Inflammatory responses involving cytokine release and chronic microglial activation have also been implicated in many CNS injuries and diseases (11–14). Progressive, chronic neuroinflammation has been observed following traumatic brain injury (TBI), a disease which is a major health concern with devastating personal and societal consequences (15–17). Chronic neuroinflammation, involving cytokine release and phagocytosis, has been linked to exacerbated pathology and progressive morbidity following TBI (18–23). Little is known, however, regarding potential alterations in non-phagocytic, physical microglial-neuronal interactions following brain trauma.

While many studies have explored the consequences of chronic neuroinflammatory changes following injury, little is known about acute alterations to physical microglial-neuronal interactions following TBI. Even less is known about the potential role that species differences could play in regulating these interactions following TBI. In 2015 the Rasband group published work demonstrating a reduction in microglia contacts on axon initial segments following TBI in rats (24). These “AXIS” microglia appeared to be ramified/non-activated. The same year we found that processes from activated microglia converged onto injured axons at 6 h following injury in a micro pig model of TBI (25). Recently, the Cullen group also found indications for microglial convergence onto injured neurons in the micro pig following diffuse brain injury (26). Specifically, they observed that microglia were in closer proximity to injured neuronal soma following TBI as compared to sham injured micro pigs. They also saw microglia in direct contact with injured neurons in multiple brain regions following injury (26). Much like our previous study, those findings showed that the microglia interacting with injured neurons appeared to be activated. These studies assessed physical interactions between different types of microglia, either ramified or active, with different neuronal components, either axon initial segments, neuronal soma or injured axonal swelling, which adds complexity. Therefore, we endeavored to explore whether the species utilized in these preclinical studies could be a potential variable contributing to these divergent findings.

We evaluated the physical interactions between microglia and injured axons acutely following a central fluid percussion injury (cFPI) in adult male rats and adult male micro pigs. We found that microglial process interactions with injured axonal swellings drastically decreased in rats post-injury. The decrease in microglial-axonal interactions was similar in both intact and injured axons in the rat and began to return to sham levels by 1 day in the injured axonal population. Contrarily, the physical neuro-immune interactions between microglia and injured axonal swellings was significantly increased compared to non-injured intact axons in micro pigs following cFPI. These data indicate that alterations in physical neuro-immune interactions following TBI are species dependent.

## Materials and methods

### Animals

Experiments were conducted in accordance with the Virginia Commonwealth University institutional ethical guidelines concerning the care and use of laboratory animals (Institutional Animal Care and Use Committee, Virginia Commonwealth University), which adhere to regulations including, but not limited to those set forth in the “Guide for the Care and Use of Laboratory Animals: 8th Edition” (National Research Council). Overall, these studies employed 12 adult (~3 months of age) male Sprague Dawley rats, weighing 400 ± 50 g (*n* = 4 for each group), that were generated as part of a previous study (25), and four adult (~6 months of age) male Yucatan micro pigs, weighing 23 ± 5 kg. Tissue from three sham-injured and four 6 h post-cFPI micro pigs, used for our previous study, were also included for the cell damage analysis (25). All animals, both rats and micro pigs, were housed in environmentally controlled rooms on a 12 h light–dark cycle, with free access to food and water and full veterinary oversight.

### Surgical preparation and injury induction

#### Rats

Anesthesia in rats was induced with 4% isoflurane and animals were then intubated and ventilated with 2% isoflurane in 30% O_2_/70% N_2_O throughout the duration of the surgery. Ophthalmic lubricant (Dechra, Overland Park, KS, USA) was applied to avoid damage or drying of the eye. Body temperature was maintained at 37°C with a rectal thermometer connected to a feedback-controlled thermoregulatory heating pad (Harvard Apparatus, Holliston, MA, USA). Heart rate, respiratory rate, and hemoglobin oxygen saturation were monitored via a hindpaw pulse oximetry sensor (STARR Life Sciences, Oakmont, PA, USA) for the duration of anesthesia to verify systemic physiology was within normal ranges. After placing rats in a stereotaxic frame (David Kopf Instruments, Tujunga, CA, USA), a midline incision was made followed by a 4.8 mm circular craniotomy, which was placed along the sagittal suture midway between bregma and lambda. The dura was left intact. The procedures used to induce cFPI were consistent with those described previously (27, 28). Briefly, a Luer-Lok syringe hub was affixed to the craniotomy site, and dental acrylic (methyl-methacrylate; Hygenic Corp., Akron, OH, USA) was applied around the hub and allowed to harden. Animals were removed from the stereotaxic frame and connected to the fluid percussion device, maintaining an unbroken fluid-filled system from the intact dura through the cylinder, via a Luer-Lok adaptor. To induce the cFPI a pendulum was released onto the fluid-filled cylinder of the cFPI device, producing a pressure pulse of 2.05 ± 0.15 atmospheres for ~23 ms, which transduced through the intact dura to the CSF. The pressure pulse was measured by a transducer affixed to the injury device and displayed on an oscilloscope (Tektronix, Beaverton, OR, USA). This injury did not result in any breach of the dura mater. Immediately after the injury, the rat was reconnected to the ventilator and the hub and dental acrylic were removed *en bloc*. Gelfoam was placed over the craniotomy/injury site, and the scalp was sutured. Rats were then allowed to recover and were returned to clean home cages. Identical surgical procedures were followed for sham-injured rats, without release of the pendulum to induce injury.

#### Micro pigs

Micro pigs were initially anesthetized with an intramuscular injection of 100 mg/ml Xylazine (2.2 mg/kg; AnaSed Injection, Shenondoah, IA, USA), 100 mg/ml Telazol (2.0 mg/kg; Tiletamine HCL and Zolazepam HCL; Pfizer, New York, NY, USA) followed by intravenous administration of sodium pentobarbital (60 mg/kg; Sigma-Aldrich, St. Louis, MO, USA). Once the absence of a corneal reflex was verified, the micro pig was intubated and ventilated with 2% isoflurane mixed in 100% oxygen to maintain anesthesia throughout the surgery. Ophthalmic lubricant was applied to avoid damage or drying of the eye. Body temperature was monitored with a rectal thermometer and maintained at 37°C with a heating pad. A small catheter was placed in the ear vein for the infusion of Lactated Ringer's solution (Hospira, Lake Forest, IL, USA) to maintain hydration. A midline incision was made from the supraorbital process to the nuchal crest and a 14 mm diameter circular craniotomy was trephined along the sagittal suture, positioning the center of the craniotomy 15 mm anterior to lambda (on the nuchal crest) and leaving the dura intact. A stainless steel custom threaded hub (Custom Design and Fabrication, Richmond, VA, USA) was screwed into the craniotomy site to a depth of ~4 mm. Screws were then placed directly posterior and anterior-lateral to the craniotomy and dental acrylic was applied around the hub and screws to insure hub stability. The procedures used to induce cFPI in the micro pig were consistent with those described above for the rat. Briefly, anesthetized micro pigs were connected to a cFPI device retrofitted with an L-shaped stainless steel adaptor that allowed for a sealed connection to the micro pig injury hub. To induce the cFPI a pendulum was released onto the fluid-filled cylinder of the cFPI device, producing a pressure pulse of pulse 1.7 ± 0.2 atmospheres for ~30 ms, which transduced through the intact dura to the CSF. The pressure pulse was measured by a transducer affixed to the injury device and displayed on an oscilloscope. Immediately after injury induction, animals were disconnected from the injury device and the dental acrylic, hub, and screws were removed *en bloc*. At no time were micro pigs taken off the ventilator. This injury did not result in any breach of the dura mater. Gelfoam was placed over the craniotomy/injury site to alleviate minute bone bleeding, and the scalp was sutured. Prior to recovery from anesthesia, micro pigs were given an intramuscular injection of 50 mg/ml Rimadyl (4.4 mg/kg; Henry Schein). Micro pigs were returned to their home pens once they regained full mobility.

### Tissue processing

At specified time points following injury (1 day for micro pigs or 6 h and 1 day for rats), animals were anesthetized, as described above, and overdosed with euthasol euthanasia-III solution (Henry Schein) followed by transcardial perfusion with 0.9% saline followed by 4% paraformaldehyde/0.2% glutaraldehyde in Millonig's buffer (135 mM sodium phosphate monobasic/109 mM sodium hydroxide) for immunohistochemical analysis. After transcardial perfusion, the brains were removed and post-fixed in 4% paraformaldehyde/0.2% glutaraldehyde/Millonig's buffer for 36–48 h. Post-fixed micro pig brains were blocked into 5 mm coronal segments throughout the rostral-caudal extent using a tissue slicer (Zivic Instruments, Pittsburgh, PA, USA). Segments of the micro pig brain containing the thalamus were bisected at the midline and the left side was analyzed. The entire rat brain and the 5 mm coronal segments containing the thalamus of the micro pig brain were sectioned coronally in 0.1 M phosphate buffer with a vibratome (Leica) to a thickness of 40 μm. Sections were collected serially in 12-well plates for rats and 6-well plates for micro pigs and stored in Millonig's buffer at 4°C.

### Evaluation of cell damage

To evaluate numbers of damaged cells in the thalamic domain of micro pigs and rats following injury, two sequential, randomly selected sections per animal (Micro pig: sham *n* = 3, 1 day *n* = 4; Rat sham *n* = 4, 1 day *n* = 4 animals) were stained with hematoxylin and eosin (H&E) and assessed as described previously (29). Briefly, tissue was mounted on gelatin-coated slides before dehydration and rehydration. Rehydrated tissue was incubated in Gills hematoxylin (Leica Biosystems, Buffalo Grove, IL, USA) followed by bluing agent (Leica Biosystems, Buffalo Grove, IL, USA) and three dips in 0.25% eosin Y/0.005% acetic acid/95% ethanol before sections were cleared through increasing concentrations of ethanol and cover-slipped with Permount (Thermo Fisher Scientific, Waltham, MA, USA). Sections were visualized using a Nikon Eclipse 800 microscope. Assessments were done on the left side of the thalamus for each section. The number of damaged neurons, delineated by eosinophilic cytoplasm and condensed nuclei, in the entire left thalamus was counted by two independent investigators blinded to the animal group and averaged for each animal and each group. Data is reported as the number of damaged cells/10 μm^2^.

### Immunohistochemistry

For visualization of diffuse axonal injury in the micro pig and rat thalamus 1 day following cFPI, sections were blocked and permeablized in 1.5% triton/10% NGS/PBS followed by overnight incubation with the primary rabbit antibody against the C-terminus of β-APP (1:700; Cat.# 51-2700, Life Technologies, Carlsbad, CA, USA) in 10% NGS/PBS at 4°C. A biotinylated goat anti-rabbit IgG (1:1,000; Cat.# BA-1000, Vector Laboratories, Burlingame, CA, USA) secondary antibody was used. The sections were then incubated in avidin biotinylated enzyme complex using the Vectastain ABC kit (Vector Laboratories, Burlingame, CA, USA) followed by visualization with 0.05% diaminobenzidine/0.01% H_2_O_2_/0.3% imidazole/PBS. The tissue was mounted, dehydrated, and cover-slipped. Visualization of APP labeled axonal swellings was performed using a Nikon Eclipse 800 microscope (Nikon, Tokyo, Japan) equipped with an Olympus DP71 camera (Olympus, Center Valley, PA, USA).

For fluorescent immunohistochemistry, micro pig and rat tissue sections were triple labeled with the following antibodies: rabbit anti-β-APP (1:700; Cat.# 51-2700, Life Technologies, Carlsbad, CA, USA), rabbit anti-Iba-1 (1:1,000; Cat.# 019-19741, Wako, Osaka, Japan), and rat anti-myelin basic protein (1:1,000; Cat.#NB600-717, Novus Biologicals, Littleton, CO, USA). For rat tissue, mouse anti-myelin MBP clone SMI-99 (1:1,000; Cat. # 808401, BioLegend, San Diego, CA, USA) was substituted for the rat anti-MBP antibody. To reduce the amount of lipid within the tissue and enhance antibody penetration, the sections were dehydrated, then rehydrated, through varying percentages of ethanol ranging from 70 to 100%, with the tissue beginning and ending in PBS. The tissue was blocked with 5% NGS/2% BSA/1.5% Triton/PBS followed by incubation with the rabbit anti-APP antibody overnight at 4°C. The tissue was then incubated with Alexa Fluor 488-conjugated goat anti-rabbit secondary antibody (1:700; Cat.# A11034, Life Technologies, Carlsbad, CA, USA), re-blocked in 5% NGS/2% BSA/PBS and incubated with rabbit anti-Iba-1 and the anti-MBP antibody (either mouse or rat). Alexa Fluor 568-conjugated goat anti-rat IgG (1:700; Cat.# A11077, Life Technologies, Carlsbad, CA, USA) or Alexa Fluor 568-conjugated goat anti-mouse IgG (1:700; Cat.# A-11031 Life Technologies) were used to visualize the MBP labeling. Alexa Fluor 633-conjugated goat anti-rabbit IgG (1:700; Cat.# A21071, Life Technologies, Carlsbad, CA, USA) was used to visualize the Iba-1 labeling. Labeled tissue was mounted using Vectashield hardset mounting medium with DAPI (Cat.# H-1500; Vector Laboratories, Burlingame, CA, USA).

### Microglia process contact analysis

The microglial process contact analysis was done as described previously (25). Briefly, Z-stacked images (10–25 μm-thick stacks; 0.32 μm between steps) were captured on a Zeiss LSM 710 confocal microscope (Carl Zeiss, Oberkochen, Germany). Regions for imaging were selected based on both the APP profile and the penetration of MBP (only the MBP penetration was used to select imaging region in sham animals). Specifically, regions with at least three APP+ swellings or MBP+ axonal segments that traversed within the section were randomly selected. Axonal swelling or MBP+ segment that were on the surface of the tissue were not selected. During selection the microglial channel was not observed. Two sections were evaluated for each animal. As the APP+ injured axons were MBP+, myelinated fibers were used as controls. Three-dimensional reconstructions of the *z*-stacks were made using Velocity software (PerkinElmer, Waltham, MA, USA). All APP+ axonal swellings contained within each 3D image were assessed in injured animals. For the assessment of intact axons or for sham-injured animals, 5–10 myelinated fibers per 3D image were chosen via random-number generator determined *x, y* coordinates. The number of myelinated fibers analyzed per 3D image depended on the penetration of MBP in the region and section. The length of the APP+ axonal swelling (distance from APP+ axonal stem to the disconnected base of APP+ swelling) or the intact myelinated MBP+ axonal segment was measured using Velocity software. Microglia process contacts were identified manually on 3D images and confirmed by stepping through the *z*-stacks. All microglial contacts within the delineated axonal segments were included in the analysis.

### Electron microscopy

To verify microglia process contacts on axonal swellings 1 day following cFPI in the micro pig, tissue was assessed for microglial process contacts as described previously (25). Briefly, a subset of tissue was immunolabeled with rabbit anti- Iba-1 (1:1,000; Cat.# 019-19741, Wako Osaka Japan) followed by incubation with biotinylated goat anti-rabbit IgG (1:1,000; Cat# BA-1000, Vector Laboratories, Burlingame, CA, USA) secondary antibody. The reaction product was visualized with 0.05% diaminobenzidine/0.01% hydrogen peroxide/0.3% imidazole in 0.1 M phosphate buffer and the tissue was prepared for EM analysis. In this approach, tissue sections were osmicated, dehydrated, and embedded in epoxy resin on plastic slides. After resin curing, the slides were studied with routine light microscopy to identify the precise thalamic areas for excision. Once identified, these sites were removed, mounted on plastic studs, and 70-nm sections were cut serially and mounted on Formvar-coated slotted grids. The grids were stained in 5% uranyl acetate in 50% methanol and 0.5% lead citrate. Ultrastructural qualitative analysis was performed using a JEOL JEM 1230 transmission electron microscope (JEOL-USA, Peabody, MA, USA) equipped with Ultrascan 4000SP CCD and Orius SC1000 CCD cameras (Gatan, Pleasanton, CA, USA).

### Statistical analysis

A Shapiro-Wilk test for normality of the data was done prior to utilizing non-parametric statistics for data that was not normally distributed. The number of axonal swellings to be assessed for each time point in each species was determined by power analysis using preliminary data, an alpha = 0.05 and a power of 80%. One-way ANOVA with Bonferroni *post-hoc* was done for cell damage analysis in both micro pig and rat thalami. Kruskal-Wallis followed by Mann-Whitney U test with a Bonferroni correction for multiple comparisons was used to compare rat histological data. Paired *t*-tests were used to compare 1 day micro pig histological data. Statistical significance was set to a *p*-value < 0.05. Data are presented as mean± standard error of the mean.

## Results

### No macroscopic pathology or overt cell death was observed in the thalamus of either the micro pig or rat following cFPI

**Figure 1:**
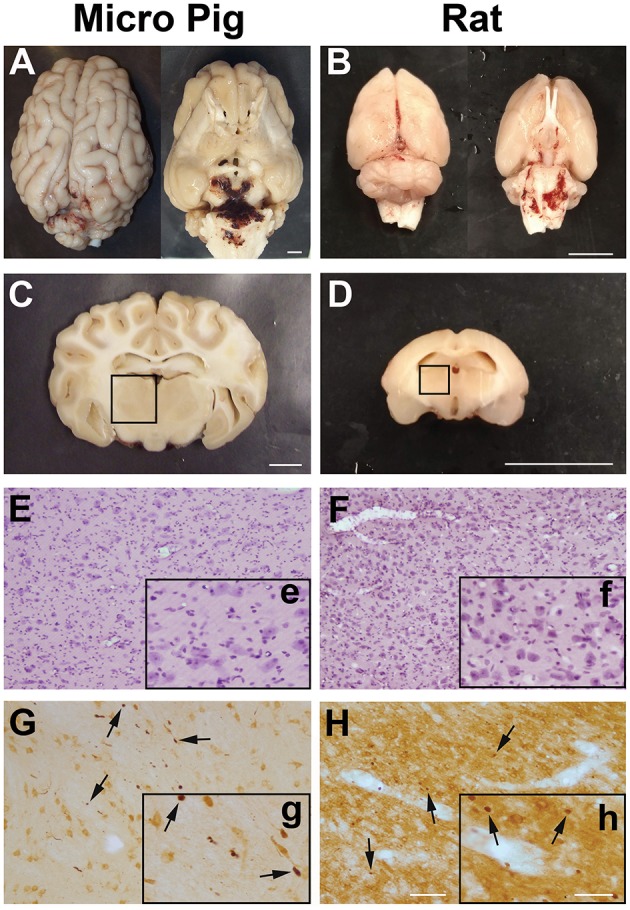
Central fluid percussion injury does not result in focal brain damage, but does precipitate diffuse axonal injury in the thalamus of both rats and micro pigs 1 day following injury. Representative photographs of the gross **(A,C)** micro pig and **(B,D)** rat brain 1 day following cFPI. The top panels are dorsal and ventral views of the entire brain 1 day post-injury while panels **(C)** and **(D)** represent 5 mm thick coronal sections at the level of the rostral thalamus in the **(C)** micro pig and **(D)** rat. The boxes indicate the regions of analysis of microglia process convergence in the thalamus of both species. Diffuse cFPI also did not result in cell damage/death. Hematoxylin and eosin staining reveled no square wave cellular damage in the thalamus of either the **(E)** micro pig or **(F)** rat thalamus at 1 day following cFPI. Amyloid precursor protein (APP) immunohistochemistry in the **(G)** micro pig and **(H)** rat thalamus, however, demonstrated diffuse axonal injury (arrows) 1 day following cFPI. Insets (e–h) depict higher magnification images of panels **(E–H)**, respectively. Note that the cFPI model employed did not result in contusion, hematoma formation or square wave cell damage/death, however did demonstrate diffusely distributed axonal injury, consistent with diffuse injury. Scale bar **A–D**: 1 mm; **E–H**: 100 um; e–h: 50 um.

As previously published, the cFPI used for both rats and micro pigs generated virtually no macroscopic pathology in either the micro pig or rat brain (Figures 1A–D) (25, 28–30). While limited subarachnoid bleeding in the caudal aspect of the brain was observed in both species 1 day following cFPI, macroscopic hemorrhage within the brain parenchyma was not detected. Importantly, while the pressure and duration of injury varied slightly between rodents and large animals, these injuries remained diffuse in nature and were not accompanied by contusion, hematoma formation or tissue loss throughout the rostral-caudal extent of either the micro pig or rat brain. There was also no discernable neuronal damage in the thalamus of either species following cFPI (F_5,18_ = 2.878, *p* = 0.044; rat sham:0.51 ± 0.21, rat 1 day post-cFPI:0.22 ± 0.06, pig sham:0.03 ± 0.003, pig 1 day post-cFPI:0.15 ± 0.05 number of damaged/dead cells/10 μm^2^, *p* > 0.05 for all Bonferroni corrected *post-hoc* comparisons, Figures 1E,F). Axonal injury, identified as APP+ axonal swellings, however, was apparent in the thalamus of both species 1 day following cFPI (Figures 1G,H). Collectively, these features speak to the diffuse nature of the injury employed in both species. Additionally, all physiological parameters measured were within the normal range throughout the surgery and following cFPI in both rats and micro pigs, consistent with previous reports (25, 30). Therefore, alterations in microglial-neuronal interactions observed could be attributed to the cFPI and not to systemic physiological changes.

### Microglial-axonal physical interactions decreased following cFPI in the rat

**Figure 2:**
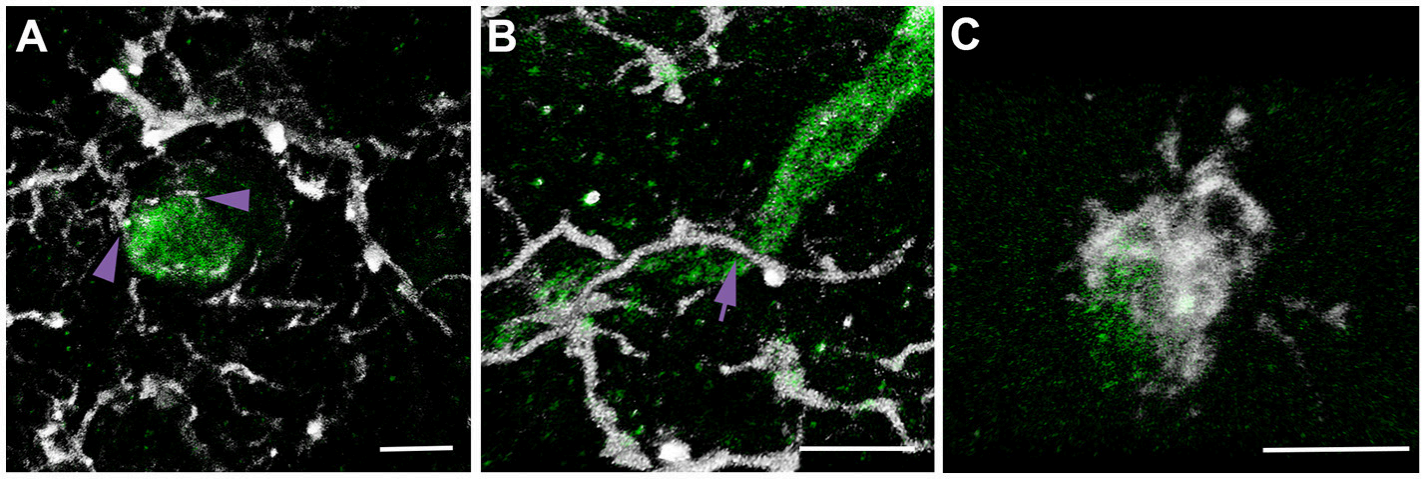
Multiple types of microglial process contacts onto axonal segments were observed following brain injury. Representative 3D reconstructions of APP+ injured axonal swellings (green) and Iba-1+ microglia (white). **(A)** Microglial processes that terminated on the axonal surface were considered “terminal processes” (purple arrow heads). **(B)** Processes that continued past the axon were considered “process passes” (purple arrow). **(C)** Processes that encircled the axon were considered “process cups.” This nomenclature and identification scheme was used for all assessments of microglial physical interactions with axons in rats and micro pigs throughout the study.

Our previous study demonstrated significant microglial process convergence onto injured myelinated fibers in the micro pig thalamus 6 h following cFPI. To assess the potential for similar microglial process convergence following TBI in the rodent thalamus, we investigated physical interactions between microglial processes and either injured APP+ myelinated axonal swellings in rats sustaining cFPI or normal myelinated fibers in sham-injured and cFPI rats. A previous study of microglial-axonal interactions in the rodent brain following TBI was done at both acute (hours) and sub-acute (days) times following injury (24), therefore we investigated the physical interactions between microglial processes and axonal segments at 6 h and 1 day post-cFPI in rats (sham *n* = 62 axonal segments; 6 h post-cFPI intact axons *n* = 60 axonal segments; 6 h post-cFPI injured axons *n* = 59 axonal swellings; 1 day post-cFPI intact axons *n* = 60 axonal segments; 1 day post-cFPI injured axons *n* = 62 axonal swellings; four rats assessed per group). This strategy allowed for assessment of a more sub-acute time point (1 day) while maintaining a time point comparison with our previous investigation of neuro-immune interactions 6 h post-cFPI in micro pigs (25). Processes were considered to be in contact with the axonal segment if the Iba-1+ process was observed in direct apposition to the surface of either the MBP+ myelin sheath of a non-injured myelinated thalamic axon, or the APP+ axonal swelling of an injured myelinated axon, in confocal 3D reconstructions. Processes that terminated on the axonal surface were considered “terminal processes,” processes that continued past the axon were considered “process passes” and processes that encircled the axon indicative of phagocytosis were considered “process cups (Figure 2).”

**Figure 3:**
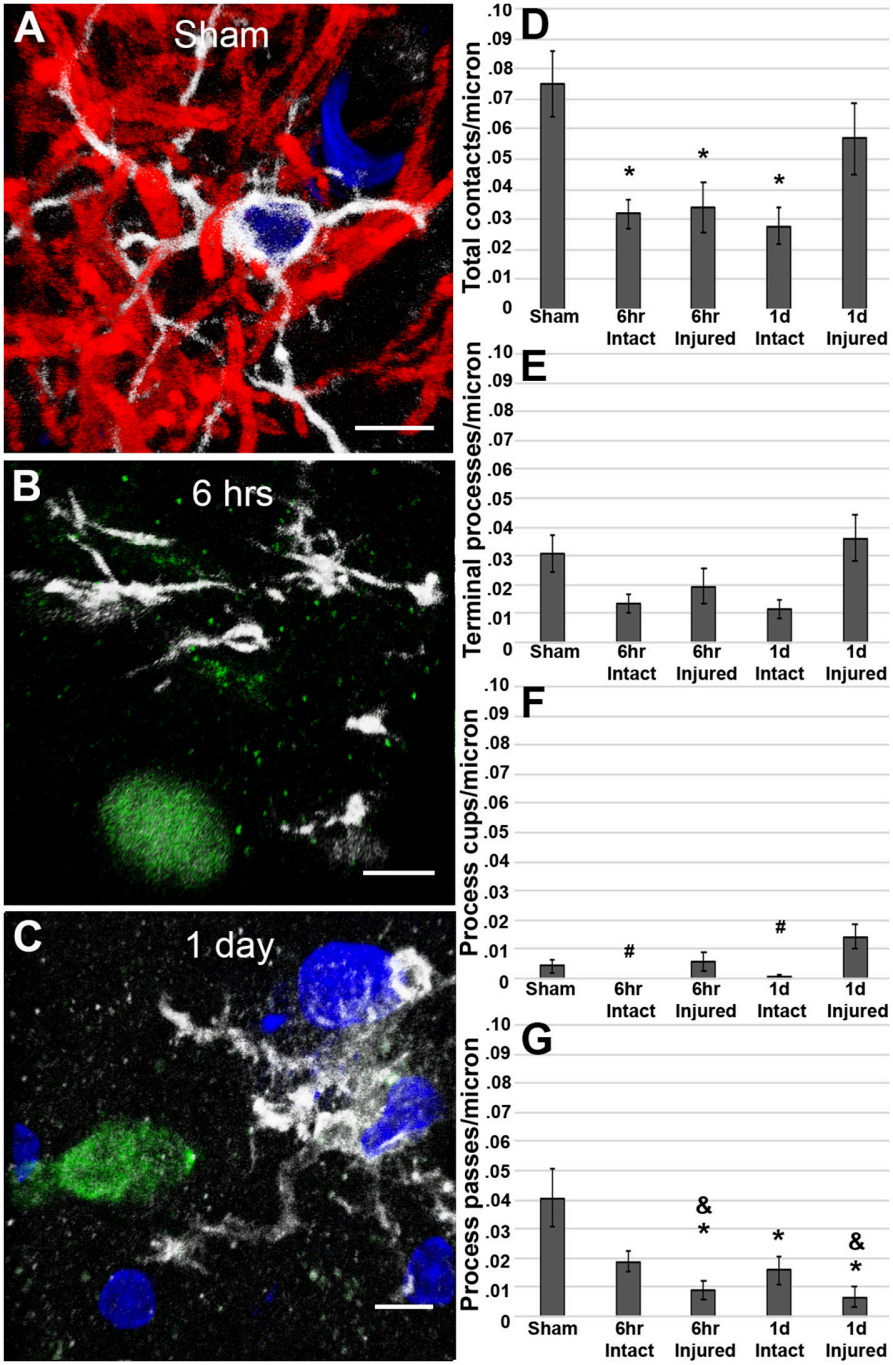
Microglial processes are less engaged with axons following cFPI in the rat. Representative 3D reconstructions of Iba-1+ microglial (white) processes with **(A)** MBP+ intact myelinated axons (red) in sham-injured rats or APP+ injured axonal swellings (green) in cFPI rats either **(B)** 6 h or **(C)** 1 day post-injury. DAPI-labeled nuclei are in blue. Bar graphs depicting the average number of **(D)** total microglial process contacts, **(E)** terminal processes, **(F)** process cups, and **(G)** process passes of Iba-1+ microglial that contact either MBP+ intact fibers or APP+ injured axonal swellings following sham or TBI in the rat. Note that overall, total microglial process contacts are decreased in intact axonal segments at both 6 h and 1 day post-cFPI and in injured axonal swellings at 6 h post-injury in rats. Graphs depict the mean ± standard error of the mean. ^*^p < 0.05 compared to sham; ^#^*p* < 0.05 compared to injured axonal swellings 1 day post-cFPI; ^&^*p* < 0.05 compared to intact axonal segments 6 h post-cFPI. Scale bar: 5 μm.

Analysis at 6 h post-cFPI showed that the number of microglial process contacts onto both injured axonal swellings and non-injured intact myelinated fibers was drastically reduced as compared to microglial processes contacting myelinated fibers in the thalamus of sham-injured rats (Kruskal-Wallis $X_{\left(4\right)}^{2}$ = 16.02, *p* = 0.002; sham vs. 6 h post-cFPI intact axonal segments *p* = 0.026; sham vs. 6 h post-cFPI injured axonal swellings *p* = 0.004; Figures 3A,B,D). While the reduction in microglial-axonal interactions was still observed in the intact myelinated fiber population 1 day following cFPI, there was no discernable difference in the number of physical microglial-axonal contacts between myelinated fibers in sham and APP+ injured axons 1 day post-cFPI in the rat (sham vs. 1 day post-cFPI intact axonal segments *p* = 8 × 10^−4^; sham vs. 1 day post-cFPI injured axonal swellings *p* = 0.232; Figures 3C,D). There were no differences in the number of microglial processes terminating around injured axonal swellings or intact axonal segments at either time point as compared to intact axons in sham-injured rats (Kruskal-Wallis terminal microglial processes $X_{\left(4\right)}^{2}$ = 9.906, *p* = 0.042, *p* > 0.05 for all Bonferroni corrected *post-hoc* comparisons; Figure 3E). There were also no drastic alterations in the number of microglial cups observed at either time point following cFPI as compared to sham [$X_{\left(4\right)}^{2}$ = 21.427, *p* = 1.3 × 10^−4^; sham vs. 6 h post-cFPI intact axonal segments *p* = 0.1, sham vs. 6 h post-cFPI injured axonal swellings *p* = 1.00, sham vs. 1 day post-cFPI intact axonal segments *p* = 0.4, sham vs. 1 day post-CPFI injured axonal swellings *p* = 0.22; Figure 3F], however, there were significantly more microglial process cups on injured axonal swellings at 1 day as compared to intact myelinated fibers at either timepoint post-cFPI, demonstrating potential for microglial phagocytosis of injured axons in the rat thalamus 1 day following cFPI (6 h post-cFPI intact axons vs. 1 day post-cFPI injured axons *p* = 8 × 10^−4^; 1 day post-cFPI intact axons vs. 1 day post-cFPI injured axons *p* = 0.001). There was, however, a significant decrease in the number of microglial processes that passed by the injured axonal swellings at both 6 h and 1 day following cFPI as compared to sham [Kruskal-Wallis $X_{\left(4\right)}^{2}$ = 25.437, *p* = 1.4 × 10^−4^; sham vs. 6 h post-cFPI injured axon *p* = 0.004, sham vs. 1 day post-cFPI injured axon *p* = 7.6 × 10^−4^; Figure 3G]. Injured axonal swellings at both timepoints post-injury had small, but significant, reductions in microglial process passes as compared to the intact myelinated axons at 6 h post-cFPI (6 h post-cFPI intact axon vs. 6 h post-cFPI injured axon *p* = 1.2 × 10^−3^; 6 h post-cFPI intact axon vs. 1 day post-cFPI injured axon *p* = 0.002). Comparisons between sham and injured axonal swellings are shown in greater detail in the Supplemental Movie Files 1–3.

### Microglial process convergence onto injured axons is increased 1 day following diffuse cFPI in the micro pig

**Figure 4:**
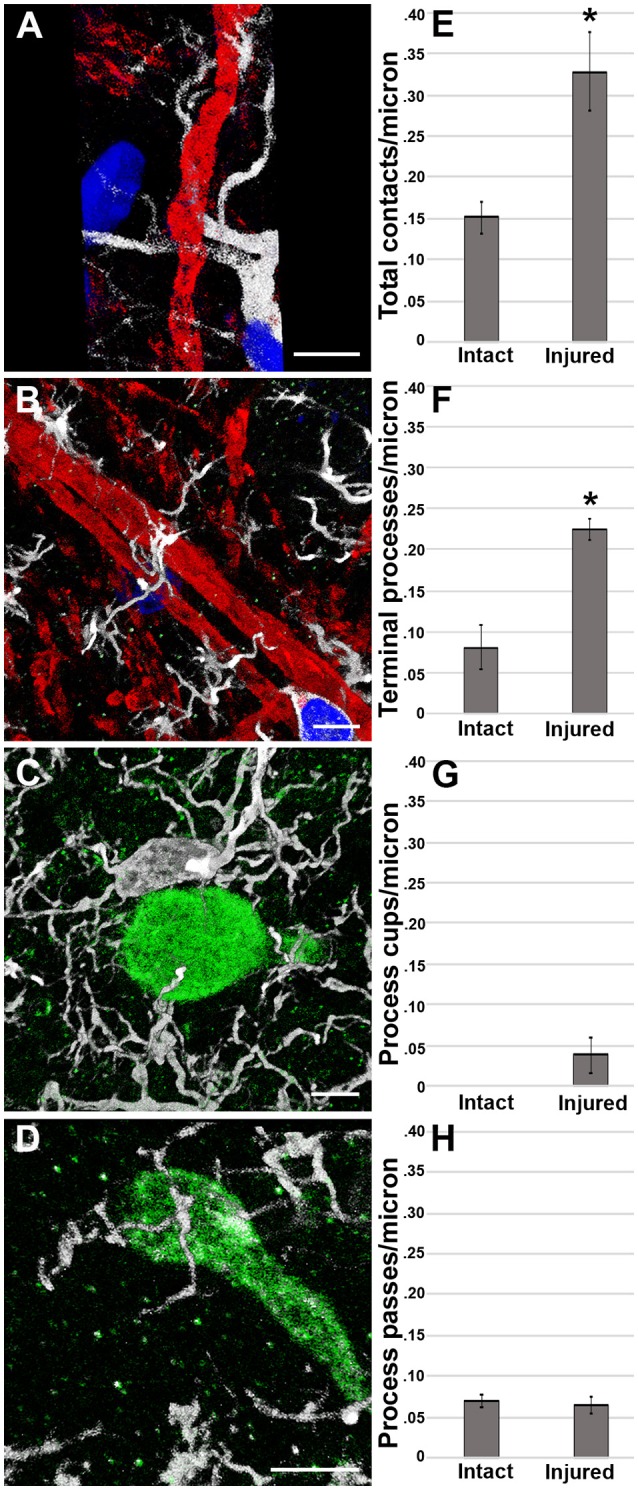
Microglial process convergence onto injured axonal swellings occurs 1 day following cFPI in the micro pig. Representative reconstructions of **(A,B)** MBP+ intact myelinated axons (red) or **(C,D)** APP+ injured axonal swellings (green) and Iba-1+ microglia (white) in the thalamus of injured micro pigs 1 day post-cFPI. DAPI-labeled nuclei are in blue. Bar graphs depicting the average number of **(E)** total microglial process contacts, **(F)** terminal processes, **(G)** process cups and **(H)** process passes of Iba-1+ microglial that contact either MBP+ intact fibers or APP+ injured axonal swellings 1 day following TBI. As previously seen, total contacts between microglial processes and injured axonal swellings was increased in the micro pig thalamus 1 day following cFPI. While there were not differences between intact and injured axonal segments in regards to microglial process passes or process cups, the increase in total process contacts was mirrored in the number of terminal processes contacting injured axonal swellings. Graphs depicts the mean ± standard error of the mean. ^*^p < 0.05. Scale bar: 5 μm.

To assess if our previous observation of microglial process convergence onto injured axonal swellings at 6 h following cFPI in the micro pig (25) persisted to a more sub-acute time point and/or progressed to phagocytosis, we analyzed microglial processes directly contacting non-injured intact axons or injured axonal swellings in the thalamus 1 day following TBI (Intact MBP+ axons *n* = 58; APP+ injured axonal swellings *n* = 58 from four micro pigs; Figure 4). Interestingly, in contrast to the results observed in the rat model, analysis of the micro pig thalamus 1 day following cFPI demonstrated a 2-fold increase in the number of total microglial process contacts onto injured axons as compared to non-injured myelinated axons (paired *t*-test *p* = 0.002; Figure 4E).

The difference in total microglial process contacts in the micro pig 1 day post-cFPI was primarily produced by the drastic increase in the number of microglial processes terminating on injured axons as compared to non-axotomized, intact axons within the micro pig thalamus 1 day post-cFPI (paired *t*-test *p* = 1.5 × 10^−5^; Figure 4F). There were very few axonal segments, either injured or intact, that were observed being cupped/encircled by microglial processes 1 day following cFPI (Paired *t*-test *p* = 0.08; Figure 4G). Additionally, the number of microglial processes passing by injured axons was similar to that of non-injured intact myelinated thalamic axons (paired *t*-test *p* = 0.77; Figure 4H). This is shown in more detail in the Supplemental Movie Files 4, 5.

**Figure 5:**
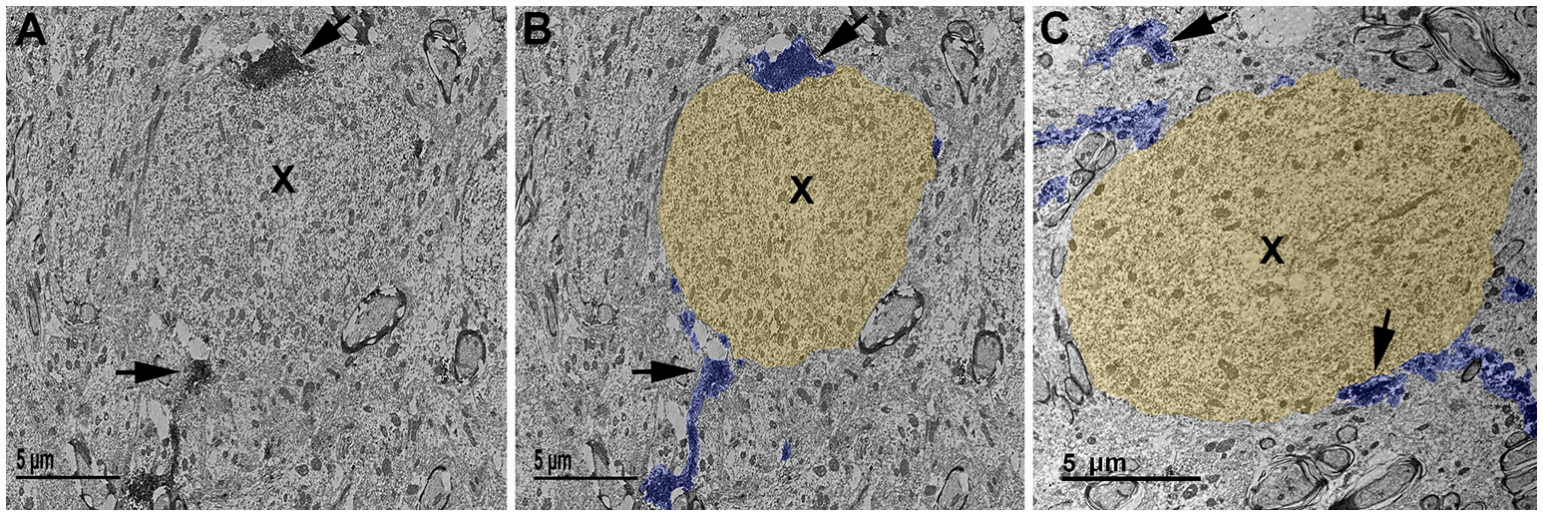
Ultrastructural evidence of direct microglial process contacts onto axonal swellings 1 day post-cFPI in the micro pig. **(A–C)** Representative electron micrographs of Iba-1 immuno-labeled microglia in the thalamus of micro pigs 1 day following cFPI. **(B,C)** Iba-1-labeled microglial processes are pseudo-colored blue and axonal swellings, as identified using common ultrastructural features observed previously following diffuse TBI, are pseudo-colored yellow for clarity. The majority of microglial processes found interacting with axonal swellings (X) 1 day post-injury appeared as immune-labeled puncta adjacent to the axonal swelling. In some cases, the microglial process could be followed for a short distance. Scale bar: 5 μm.

Furthermore, to verify that the microglial processes visualized to be in contact with injured axonal swellings at the confocal level were in direct apposition to the plasmalemma of axonal swellings 1 day post-TBI in the micro pig thalamus, these interactions were qualitatively assessed at the ultrastructural level. Injured axonal segments were identified by their ultrastructural characteristics, including clumped disordered neurofilaments and organelle accumulation. Identified axonal segments did not, however, have features that are indicative of Wallerian degeneration, such as vacuolization or lucent zones (25). As depicted in Figure 5, Iba-1 labeled microglial processes made direct contact with the plasma membrane of axonal swellings in the micro pig thalamus 1 day following cFPI. Consistent with the confocal assessment of microglia cups, these contacts, did not appear to be associated with phagocytic engulfment of the axonal swelling.

## Discussion

The current study demonstrates that the species utilized for *in vivo* pre-clinical studies influences the manner in which microglial-axonal interactions change following TBI. Using quantitative image analysis of 3D confocal micrographs, we found that microglial process interactions with injured axonal swellings drastically decreased in rats post-injury. However, interactions between microglia and injured axonal swellings significantly increased in micro pigs post-injury, indicative of microglial process convergence without evidence of phagocytosis. To our knowledge, this is the first direct comparison of microglial-axonal physical interactions between rats and micro pigs following brain injury.

Using quantitative multiplexed 3D confocal image analysis, we found that microglial process convergence onto injured axonal swellings persisted 1 day following TBI in the micro pig thalamus. Specifically, there were twice the microglial terminal processes converging onto injured axonal swellings 1 day following injury compared to our previous observations at 6 h following cFPI in the micro pig (25). Interestingly, the non-injured intact axonal segments at 1 day post-cFPI also demonstrated increased microglial process convergence as compared to the previously reported sham levels of microglial process interactions (25). The degree of process convergence onto these intact axonal segments at 1 day post-injury were also similar to our previous observations for the injured axonal population at 6 h following injury in the micro pig. This could indicate a more universal increase in microglial-neuronal interaction that progresses following diffuse TBI, regardless of axonal injury. Since the axonal paths within the thalamus are not linearly arranged, this similarity between 6 h injured and 1 day intact axons could also reflect microglial process convergence onto injured axons in which the APP+ swelling is out of the plane of section. As there was a significant increase in the number of microglial process contacts onto injured axons as compared to intact, non-injured axonal segments in the micro pig thalamus 1 day post-cFPI, it appears that microglial process convergence is, however, specific to injured axons and is not likely to be merely a universal microglial response to TBI in the micro pig. Similar to our previous findings at 6 h in the micro pig, the physical interactions between microglia and injured axons at 1 day post-injury were not consistent with engulfment via microglial phagocytosis in micro pigs (10, 31, 32). As many studies have demonstrated that phagocytic activation of microglia can occur within minutes of injury (33, 34), our findings indicate that post-injury microglial process convergence is not merely a pre-phagocytic phenomenon, but may be playing a different role in the micro pig. While the potential role of microglial terminal process convergence remains tenuous, the lack of microglial process cups at 1 day post-injury could indicate a protective effect, as speculated previously (35).

The number of microglial process contacts per axonal segment in the micro pig was consistently 10-fold higher than that of the rat, regardless of injury. This remarkable difference could be due to differences in the biomechanical response to cFPI in lissencephalic verses gyrencephalic brains. Specifically, when employing an acceleration-induced brain injury, significantly greater force is required to produce similar levels of damage in lissencephalic animals with smaller brains as compared to higher order gyrencephalic species (36). While, we are still very early in our exploration of the pathobiology produced by cFPI in the gyrencephalic micro pig brain, we did find that diffuse thalamic damage without additional focal lesion was consistently produced in both species. There is also a potential that the differences in biomechanical response to injury between rats and micro pigs could result in differences in injury severity that might also alter microglial-neuronal interaction in the face of cFPI. Further studies, however, would be needed to evaluate potential confounds of slight differences in injury severity and/or biomechanical/structural differences between rats and micro pigs and how those potential differences might influence injury-mediated changes in microglial-axonal interactions.

Difference in overall microglial-axonal interactions could also reflect lower numbers of microglia in rats as compared to higher order animals, like micro pigs. In fact, dogma states that the glia/neuron ratio (or gray-white matter ratio) in lissencephalic species, such as rats, is smaller than that in gyrencephalic speices, such as primates and humans, in which many more glia per neuron are present (37). Recently, however, much of this has been called into question when more recent assessments found very little difference in the glia/neuron ratio between rats and primates (37–39). The studies regarding glia/neuron ratio in pigs determined that each strain of pig may be widely divergent in terms of the ratio of glia to neurons (40), and the more recent studies on glia/neuron ratios in various species did not assess micro pigs. Therefore, rigorous assessments of glia/neuron ratios in micro pigs compared to rats would be needed in order to evaluate potential influences of glia/neuron ratios on microglial-neuronal interactions in different species.

Remarkably, in rats we found that the physical interactions between microglia and injured axonal swellings decreased by half at 6 h following diffuse cFPI as compared to normal myelinated thalamic axons in sham controls, however, was comparable to sham at 1 day post-cFPI. The reduction in microglial process interactions compared to sham-injured controls was similarly observed in the non-injured intact axonal population at both timepoints following TBI in the rat, indicative of a general reduction in microglial interactions with axons following diffuse injury. Injured axonal swellings 1 day post-cFPI did have significantly more microglial process cups as compared to intact axonal segments at either post-injury timepoint, suggesting that, unlike in the micro pig, microglial processes terminating on injured axonal swellings may be a precursor to phagocytosis. Finally, there was also a small, but significant, reduction in microglial processes passing by injured axonal swellings as compared to non-injured intact axonal segments at 6 h post-cFPI, while no differences were observed in the number of microglial processes terminating onto axonal segments in any condition in the rat. While little is known about the structure/function of microglial process passes, the drastic reduction in microglial processes passing over axonal segments in the rat, may reflect the morphological remodeling of the microglia following injury, as activated microglia have shorter processes and less complex process networks overall. The lack of change in the number of microglial process passes in the micro pig model may indicate that the morphological changes of activated microglia in the micro pig progress over a longer period of time following injury, however these speculations would require proper investigation in follow-up studies. Taken together, the rat data suggest that rodent microglia have the opposite response to TBI-induced axonal injury as compared to the micro pig and are either non-responsive to or disengage from injured axons acutely following brain injury.

This contradictory response to axonal injury acutely following diffuse TBI could be suggestive of various potential differences between micro pig and rat microglia. One possibility revolves around the variation in anesthesia and analgesics given to micro pigs as compared to rats. In the current study, there were differences in the anesthesia used for induction between rats and micro pigs, which could alter inflammation and pathological response to injury given the well-known effects of isoflurane in this regard (41–47). However, the maintenance anesthesia during surgery was consistently isoflurane for both species, reducing this likelihood. Additionally, in the current study Rimadyl (a non-steroid anti-inflammatory drug; NSAID) was administered to micro pigs as an analgesic, but was not administered to rats post-injury. We did, however, also observed substantial microglial process convergence at 6 h post-cFPI in non-survival micro pigs, in which Rimadyl was not administered (25). There was also a 2-fold increase in microglial process contacts onto injured axonal swellings as compared to intact non-injured axons 1 day post-cFPI in the micro pig. Finally, micro pigs within the Wofford study were not administered NSAIDs, but microglial process convergence was still observed following TBI (26). Therefore, it is unlikely that microglial process convergence seen in the micro pig following cFPI is merely a side-effect of Rimadyl treatment; however, additional studies would be required to assess the role of NSAIDs in microglial process convergence following TBI.

Another intriguing possibility is that differences in microglial process interactions between rats and micro pigs reflect potential differences in the dynamics and/or signaling mechanisms of microglial processes between the two species. Rodent microglia are capable of process convergence onto neurons, as seen in other disease states (48–50). Epilepsy is one such disease in which several groups have shown microglial process convergence onto hyperactive neuronal segments (49–51). These interactions appear to serve a potentially protective function by decreasing the likelihood of excitotoxicity in overly active neurons (51) and appear to be controlled by fractalkine and/or glutamate signaling (52). While the mechanism of microglial process convergence following TBI in the micro pig might be different than those explored in epileptic rodents, these studies demonstrate that microglial process convergence does occur in rodents and that molecular mechanisms for microglial process convergence could potentially be conserved across vertebrate species. Ultimately, the divergence in microglial process response to axonal injury following TBI in rats and micro pigs can be leveraged to greatly expand our understanding of microglial-neuronal physical interactions and the consequences of these interactions on the pathophysiology of TBI-induced axonal injury.

The current study focused on microglial-axonal physical interactions in pre-clinical models, however, there are reports indicating that microglia physically interact with injured axons following TBI in the human brain. In a 2014 study, Ryu et al. demonstrated co-labeling of microglial Iba-1 with APP+ axonal swellings in brains of veterans who had histories of blast injury exposure (53). Another study, by Oehmichen et al. showed potential microglial process convergence onto injured axonal swellings when employing double-labeling techniques in human TBI tissue (54). These studies give an indication that microglial processes may contact axonal swellings in the human brain following TBI; however, further investigation is needed to comprehensively assess potential alterations in microglial-neuronal physical interactions in the human population and address how those changes compare to those observed pre-clinically. Additionally, while one study demonstrated that neuronal regeneration is positively correlated to increased microglial density following TBI in clinical samples (55), the role of the microglial-axonal interactions evaluated in the current study, as either detrimental or ameliorative, remain to be determined.

## Conclusions

Alterations in microglial-axonal interactions acutely following diffuse TBI appear to be, at least in part, species dependent. Following brain injury microglial processes converge onto injured axonal swellings in micro pigs. Conversely, there is a reduction in interactions between microglial processes and both intact and injured axons in rats following TBI. The divergent responses of rodent and micro pig microglia to TBI-induced axonal injury can be exploited to further our understanding of neuronal-microgilal physical interactions and the potential role these interactions play in the pathophysiological progression of diffuse axonal injury.

## Author contributions

KG processed and carried out the analysis of microscopic assessments and wrote the manuscript. AL carried out the confocal microscopic and ultrastructural analyses, conceived, designed and coordinated the study, and wrote the manuscript.

### Conflict of interest statement

The authors declare that the research was conducted in the absence of any commercial or financial relationships that could be construed as a potential conflict of interest.
