# Supplementary material for: The Importance of Inter-Species Variation in Traumatic Brain Injury-Induced Alterations of Microglial-Axonal Interactions
Source: Front Neurol. 2018 Sep 20;9:778. doi: 10.3389/fneur.2018.00778 (PMC6158363; doi:10.3389/fneur.2018.00778)
Supplement: Supplementary file 6 [file Data_Sheet_1.pdf]

Supplementary Material

The importance of intra-species variation in traumatic brain injury-induced alterations of microglial-axonal interactions.

Karen M. Gorse[1] and Audrey D. Lafrenaye[1*]

*** Correspondence:** Audrey D Lafrenaye, Ph.D.; Email: audrey.lafrenaye@vcuhealth.org

1.1 Supplementary Figures

Supplementary Movie 1 Processes of ramified/resting microglia primarily pass over normal myelinated fibers in the thalamus of rats following sham injury. Movie representing 3D reconstructions of confocal z-stacks taken from sham-injured rat thalami. DAPI labeled nuclei are in blue, MBP+ myelinated axons are in red and Iba-1+ microglia are in white. Purple circles highlight contacts between microglial processes and normal myelinated fibers. Notice that very few microglial processes come in direct contact with myelinated fibers in the rat following sham injury and that most of these microglial contacts are from processes passing by the myelinated fiber.

Suplementary Movie 2 There is a reduction in microglial process contacts onto injured axons in the rat thalamus 6 hours following cFPI. Movie representing 3D reconstructions of confocal z-stacks of thalamic sites from cFPI rats, in which APP+ injured axonal swellings are in green, Iba-1+ microglia are in white, DAPI labeled nuclei are in blue and MBP+ myelinated axons are in red. Purple circles highlight contacts between microglial processes and APP+ axonal swellings. Physical microglial process interactions with injured axonal swellings in the rat thalamus were scarce 6 hours following cFPI.

Supplementary Movie 3 Microglial process contacts onto injured axons remain reduced 1 day after cFPI in the rat thalamus. Movie representing 3D reconstructions of confocal z-stacks of thalamic sites from cFPI rat day post-injury. APP+ injured axonal swellings are in green, Iba-1+ microglia are in white, Dapi labeled nuclei are in blue and MBP+ myelinated axons are in red. Purple circles highlight contacts between microglial processes and APP+ axonal swellings. Note that microglial processes coming into contact with APP+ axonal swellings remained rare 1 day following cFPI in the rat.

Supplementary Movie 4 Microglial processes primarily pass over normal myelinated fibers in the injured micro pig thalamus. Movie representing 3D reconstructions of confocal z-stacks in the micro pig thalamus from cFPI animals 1 day post-injury. DAPI labeled nuclei are in blue, MBP+ myelinated axons are in red and Iba-1+ microglia are in white. Purple circles highlight contacts between microglial processes and normal myelinated fibers. Notice that only a few microglial processes come in direct apposition with normal myelinated fibers and that the majority of these microglial processes that do contact myelinated fibers pass over the myelinated fiber.

Supplementary Movie 5 Microglial processes converge onto injured axons 1 day following cFPI in the micro pig thalamus. Movie representing 3D reconstructions of confocal z-stacks taken in regions demonstrating axonal damage within the micro pig thalamus 1 day following cFPI. Injured, APP+ axonal swellings are in green, Iba-1+ microglia are in white, MBP+ myelinated axons are in red and DAPI labeled nuclei are in blue. Purple circles highlight contacts between microglial processes and APP+ injured axonal swellings. Note that the majority of microglial processes that come into contact with APP+ axonal swellings terminate on the proximal swelling, indicating process convergence.

frontiers
